# Retrospective Analysis of the Integration of Palliative Care Into the Care of Stroke Patients Admitted to a Regional Stroke Center

**DOI:** 10.1177/10499091241253538

**Published:** 2024-05-09

**Authors:** Houman Khosravani, Meera Mahendiran, Brindan Sivanandan, Sandra Gardner, Gustavo Saposnik, Jahnel Brookes, Anna Berall, Giulia-Anna Perri

**Affiliations:** 1 Hurvitz Brain Sciences Centre, 71545 Sunnybrook Health Sciences Center, Toronto, ON, Canada; 2 Neurology Quality and Innovation Lab (NQIL), Division of Neurology, 204352 University of Toronto Faculty of Medicine, Toronto, ON, Canada; 3 Department of Family and Community Medicine, 204352 University of Toronto Faculty of Medicine, Toronto, ON, Canada; 4 Biostatistics Division, Dalla Lana School of Public Health, 204352 University of Toronto, Toronto, ON, Canada; 5 Clinical Outcomes and Decision Neuroscience Research Center, Li Ka Shing Knowledge Institute, St. Michael’s Hospital, Toronto, ON, Canada; 6 Rotman Research Institute, Baycrest Academy for Research and Education, Toronto, ON, Canada; 7 Division of Palliative Care, Department of Family and Community Medicine, Baycrest Health Sciences, University of Toronto Faculty of Medicine, Toronto, ON, Canada

**Keywords:** end of life, stroke, stroke education, palliative care, neurology specific areas of palliative care, intensive care unit/critical care issues in palliative care

## Abstract

**Background:** Palliative care (PC) aims to enhance the quality of life for patients when confronted with serious illness. As stroke inflicts high morbidity and mortality, the integration of PC within acute stroke care remains an important aspect of quality inpatient care. However, there is a tendency to offer PC to stroke patients only when death appears imminent. We aim to understand why this may be by examining stroke patients admitted to a regional stroke centre who subsequently died and their provision of PC. **Methods:** We conducted a retrospective single-centre cohort study of patients who died during admission to the regional stroke centre at Sunnybrook Health Sciences Centre (SHSC) in Toronto, Ontario, Canada. Baseline demographics were assessed using means, standard deviations (SD), medians, interquartile ranges (IQR), and proportions. Descriptive statistics, univariate, and multivariate analyses were performed to ascertain relationships between collected variables. **Results:** Univariate modeling demonstrated that older age, being female, no stroke diagnosis at admission to hospital, ischemic stroke, and comorbidities of cancer or dementia were associated with a higher incidence of palliative medicine consultation (PMC), while admission from an acute care hospital and a Glasgow Coma Scale (GCS) coma classification were associated with a lower incidence of PMC. The multivariate model identified the GCS coma-related category as the only significant factor associated with a higher incidence of death but was non-significantly related to a lower incidence of PMC. **Conclusion:** These results highlight continued missed opportunities for PC in stroke patients and underscore the need to better optimize PMC.

## Background

The impact of a stroke is noted across the globe, with more than 15 million people experiencing a stroke per annum worldwide.^1^ Stroke remains the second main cause of death worldwide and the third main cause of death in Canada.^1,2^ Although there have been extraordinary advances in recent years in the treatment and prevention of stroke, most survivors continue to have profound disabilities and reduced quality of life.^3-5^ In Canada alone, stroke is the tenth largest contributor to disability-adjusted life years.^6^

With this significant mortality and morbidity across the illness trajectory, it follows that quality stroke care includes palliative care, which is supported by the American Heart Association/American Stroke Association policy statement.^7^ Overarching goals of palliative care include enhancing the quality of life of patients and their families facing such life-threatening illnesses by providing management of pain symptoms along with physical, psychosocial, and spiritual needs.^8^

Unlike other chronic illnesses for which palliative care is more established, the illness trajectory of stroke is unique and not always foreseeable. In situations of severe stroke, there can be acute neurological devastation with only a narrow time window in which injury reversal is possible. In these medically complex scenarios, acute stroke patients and their families have established palliative care needs that are immediate, common, and significant.^9-11^ The literature has established the immense benefits of early palliative care integration in patients with malignancy - these include improved quality of life, better symptom control, increased patient and caregiver satisfaction and more appropriate health resource use.^12,13^ As such, it is plausible that restorative care and palliative care can work in tandem when caring for patients with stroke. Yet, while there is emerging interest in the early integration of palliative care into acute stroke care through a primary stroke interdisciplinary team, there remains a tendency to offer palliative care to stroke patients only when death appears imminent, with consultation with palliative care specialists only occurring at end-of-life decisions.^7,14,15^

Currently, there is a paucity of data on stroke palliative care for patients admitted to regional stroke centers, also known as comprehensive stroke centers. Specifically, little is known about the timing and scope of palliative care provision for such patients in this setting. Therefore, the aim of this study is to examine stroke patients admitted to a regional stroke center that subsequently died and the concomitant provision of palliative care to these patients. We will examine the demographic and clinical covariates associated with the provision of palliative medicine consultation (PMC).

## Methods

### Study design and population

We conducted a retrospective single-centre cohort study of patients who died during admission to the Regional Stroke Center at Sunnybrook Health Sciences Center (SHSC) in Toronto, Ontario, Canada. The study was granted research ethics approval by the Sunnybrook Research Ethics Board, approval number 2535. At the time of data collection, SHSC remained a tertiary care centre capable of hyperacute stroke therapies including thrombolysis with tissue plasminogen activator (TPA) administration and endovascular clot retrieval (EVT), as well as the ability to offer inpatient palliative care consultation and transfer to a dedicated palliative care unit.

Our study reviewed the charts of patients that died with a underlying stroke diagnosis. Thus, patient who died were reviewed with the chart consisting of a total of 320 patients spanning January 2nd, 2016, to February 28th, 2020. Eligible patients were patients with a diagnosis of stroke (both ischemic and hemorrhagic; as defined by the International Classification of Diseases, 10th Revision Codes (ICD-10) at presentation to, or during admission to hospital, with a known subsequent death date during or immediately after admission. Patients with a diagnosis of stroke on admission were admitted with stroke as the designated service, to various locations including inpatient neurovascular units/stroke units, neuro-observations units, and other critical care units.

### Data collection

Chart review of electronic medical records over a 4-year period was assessed for patients who died with an underlying diagnosis of stroke (ischemic and hemorrhagic). Baseline demographics were collected, as well as data and sources of admission (home, transfer from acute care hospital, long term care), stroke type, stroke at presentation to hospital compared to post-admission, and code status at admission. Comorbidities captured included previous stroke history, cancer and dementia history. Severity of stroke was noted with post-stroke NIH Stroke Scale (NIHSS; Low 0-7, Moderate 8-15, Severe 16+), post-stroke Alberta Stroke Program Early CT Score (ASPECTS; 0-5, 6-8, 9-10), and post-stroke Glasgow Coma Scale (GCS; 3-8 vs 9-15). In many cases, due to stroke severity, the GCS was used in the clinical chart to document the patient’s status. A combined categorization called severe stroke was created based on NIHSS and GCS and was designated if a patient had an NIHSS >16 or GCS ≥8. Stroke treatment received was subdivided into those that received no therapy after diagnosis of stroke, medical management only, hyperacute treatment (TPA or EVT), or any neurosurgical intervention +/− hyperacute treatment. The date and location of death and date of the first palliative care consultation, if it occurred, were also collected.

### Analysis Methods

Baseline demographics were assessed using summaries of the data including means, standard deviations (SD), medians, interquartile ranges (IQR), and proportions.

Outcome analysis was conducted using competing risk survival analysis with the outcome of interest designated as the time to palliative care consultation from the admission date. Death occurring prior to palliative care consultation was set as a competing event (precludes the outcome of interest) setting the framework for the competing risk survival analysis. Patients (n = 7) who died in a post-acute hospital were censored as of their discharge date. Each competing risk outcome was initially summarized using non-parametric Kaplan-Meier (KM) cumulative incidence functions for competing risks (CIF). Fine and Gray proportional hazard subdistribution regression modeling was used for both univariate and multivariate analyses.^16^ These models estimate the subdistribution hazard ratios of the categorized demographic and clinical covariates or per unit increase in a continuous covariate. Multivariate Fine and Gray models were developed using a selection of the baseline demographic and clinical covariates. Time varying covariates, interaction terms, and non-linear parameterization were not considered.

The data analysis for this report was generated using SAS/STAT software version 15.2 and the SAS System for Windows version 9.4. Copyright © 2016 SAS Institute Inc.

## Results

### Demographics and clinical characteristics

The study cohort (n = 320) had a mean age of 72.7 years (SD = 15.6, Table 1), and almost an even split between male (50.9%) and female (49.1%) patients. Slightly more than half of patients were admitted from community settings (56.4%, n = 319), while an additional third (38.6%) were transferred from other acute care hospitals. Most patients were presented with a stroke diagnosis upon admission (88.4%), while a small portion were diagnosed post-admission (11.6%). Ischemic strokes were observed in 54.2% of cases (n = 319), with hemorrhagic strokes accounting for the remaining 45.8%. Most patients experienced their first stroke during the study period (79.6%, n = 318), with the remaining having had a recurrent stroke.

**Table 1.** Demographic and Clinical Characteristics of Study Population.

Demographic and Clinical Characteristics	n	Mean (SD)
Age	320	72.7 (15.6)
Length of stay (days)^a^	320	6 (2.14)
Gender (n = 320)		
Male	163	50.9
Female	157	49.1
Admission type (n = 319)		
Acute care hospital	123	38.6
Home in the community	180	56.4
Other	16	5.0
Stroke at admission (n = 320)		
No	37	11.6
Yes	283	88.4
Stroke type (n = 319)		
Ischemic stroke	173	54.2
Hemorrhagic stroke	146	45.8
Stroke history (n = 318)		
First stroke	253	79.6
Recurrent stroke	65	20.4
Cancer comorbidity (n = 317)		
No	259	81.7
Yes	58	18.3
Dementia comorbidity (n = 318)		
No	277	87.1
Yes	41	12.9
Code status at admission (n = 282)		
Full code	156	55.3
DNR	126	44.7
NIHSS at admission (n = 150)		
0-7 low	26	17.3
8-15 moderate	32	21.3
16+ severe	92	61.3
ASPECTS at admission (n = 85)		
0-5	23	27.1
6-8	41	48.2
9-10	21	24.7
GCS at admission (n = 268)		
3-8 - coma	129	48.1
9-15	139	51.9
Severe stroke (NIHSS 16+ or GCS ≤8, n = 300)		
No	112	37.3
Yes	188	62.7
Stroke treatment category (n = 320)		
No stroke treatment	41	12.8
Medical management only	142	44.4
Any hyperacute treatment (tPA +/− EVT)	79	24.7
Neurosurgical intervention	58	18.1
Palliative medical consult (n = 320)		
No	228	71.3
Yes	92	28.8
Location of death (n = 320)		
Other ICU	193	60.3
Stroke ward	60	18.8
Stroke ICU	32	10.0
Other ward	23	7.2
Palliative care unit	5	1.6
Post-acute care hospital	7	2.2

^a^Not adjusted for time to consult.

In terms of stroke severity, more than 60% of patients (61.3%, n = 150) had an NIHSS >16 post-stroke. Additionally, ASPECTS analysis revealed that 27.1% of patients (n = 85) had the greatest extent of ischemic changes on presentation. The GCS scores showed a balanced distribution of patients between the low GCS group (GCS 3-8; 48.1%, n = 268) and the high GCS group (GCS 9-15; 51.9%). In combination, many strokes in this study were categorized as severe strokes (GCS ≥8, or NIHSS >16; 62.7%, n = 300).

In addition, regarding comorbidities, 18.3% (n = 317) of patients had a history of cancer and 12.9% (n = 318) had a history of dementia (n = 318). The DNR code status was noted in 44.7% of cases (n = 282). Palliative care consultation was provided to 28.8% patients, without adjusting for the length of stay and competing risk of death. With regards to treatment, 12.8% did not receive any dedicated stroke treatment post-stroke. Medical management alone was administered to 44.4% of the patients, while hyperacute therapies (including TPA and endovascular therapy) were given to 24.7% of the patients. Additionally, neurosurgical intervention +/− hyperacute therapy were offered to 18.1% of patients.

The majority of stroke patients (n = 193, 60.3%) died in intensive care units other than the stroke intensive care unit, 18.8% died on stroke units (n = 60), 10.0% died in the stroke intensive care unit (n = 32), 7.2% died on other units (n = 23), 2.2% died in a post-acute care hospital, and 1.6% died in the PCU (n = 5).

The mean length of stay/time to death for the study population (n = 320) was 16 days [SD = 40.7; median = 6 days (IQR 2, 14)] with the mean timing for a PMC (n = 92) 13.7 days [SD = 21.4; median = 6 days (IQR 2, 14)].

### Competing risks

**Figure 1.**
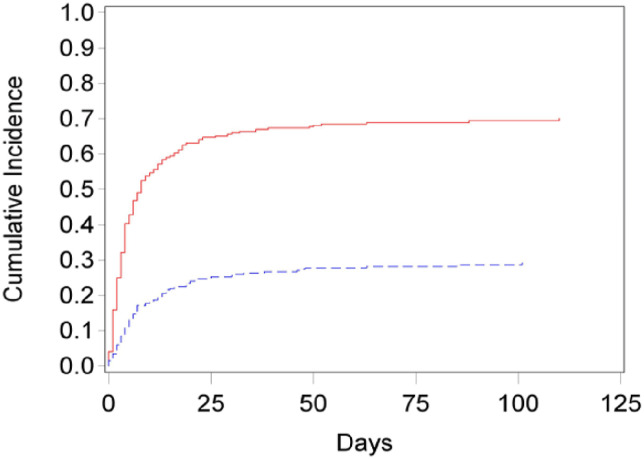
Cumulative incidence functions for the competing risks. There is a lower cumulative incidence of palliative care consultation (blue dashed line) when compared to the incidence of death (red solid line) over time.

Figure 1 illustrates the cumulative incidence functions for the competing risks. There is a lower cumulative incidence of palliative medicine consult (PMC) when compared to the incidence of death over time.

### Univariate modelling

Older age (*P* < .0011, Table 2), absence of stroke diagnosis at admission (*P* = .001), ischemic stroke (*P* < .0001), comorbidity of cancer (*P* = .0003) or dementia (*P* = .002) were each associated with a reduced incidence of the competing risk of death. Conversely, admission from an acute care hospital (*P* = .02) and GCS coma classification (*P* < .0001) were each associated with a higher incidence of the competing risk of death. Older age (*P* < .0001), female gender (*P* = .02), absence of stroke diagnosis at admission (*P* = .02), ischemic stroke (*P* = .0002), comorbidity of cancer (*P* = .0004) or dementia (*P* = .005) were each associated with a higher incidence of the competing risk of PMC. Conversely, admission from an acute care hospital (*P* = .04) and GCS coma classification (*P* = .002) were each associated with a lower incidence of the competing risk of PMC. Notably, some covariates, including admission source, demonstrated evidence of non-proportional hazards for one or both competing risks (results not presented).

**Table 2.** Univariate Fine and Gray Model – Competing Risk Analysis.

Covariate	n	Value	Death in Hospital				Palliative Care Consult			
			HR^a^	95% CI		*P* ^†^	HR^a^	95% CI		*P* ^†^
Age (per year)	320	Older age	.98	.97	.99	<.0001	1.04	1.02	1.06	<.0001
Gender (ref male)	320	Female	.81	.63	1.05	.1059	1.64	1.09	2.47	.0174
Admission source (ref home in the community)	319	Acute care hospital	2.11	1.63	2.73	<.0001	.31	.18	.54	<.0001
		Other	.81	.39	1.68	.5710	1.60	.74	3.44	.2305
Stroke at admission (ref yes)	320	No	.53	.36	.78	.0012	1.81	1.11	2.94	.0179
Stroke type (ref hemorrhagic stroke)	319	Ischemic stroke	.53	.41	.68	<.0001	2.33	1.50	3.63	.0002
Stroke history (ref first stroke)	318	Recurrent stroke	.79	.56	1.10	.1624	1.27	.79	2.03	.3283
Cancer comorbidity (ref no)	317	Yes	.51	.35	.74	.0003	2.16	1.41	3.32	.0004
Dementia comorbidity (ref no)	318	Yes	.47	.29	.76	.0021	2.02	1.24	3.31	.0050
Code status at admission (ref full code)	282	DNR	1.10	.83	1.47	.5117	1.30	.86	1.96	.2210
NIHSS at admission (ref 0-7 low)	150	8-15 moderate	1.01	.54	1.89	.9759	1.10	.51	2.37	.8092
		16+ severe	1.10	.63	1.90	.7427	1.20	.63	2.31	.5825
ASPECTS at admission (ref 9-10)	85	0-5	.72	.36	1.43	.3474	1.37	.49	3.85	.5525
		6-8	.63	.33	1.19	.1525	1.60	.63	4.09	.3258
GCS at admission (ref 9-15)	268	3-8 - coma	1.76	1.35	2.30	<.0001	.44	.26	.74	.0022
Severe stroke (ref no)	300	Yes	1.25	.96	1.63	.1010	.99	.64	1.52	.9563
Stroke treatment category (ref medical management)	320	Any hyperacute treatment	1.70	1.21	2.40	.0025	.71	.44	1.16	.1756
		Neurosurgical intervention	2.42	1.80	3.26	<.0001	.18	.07	.43	.0001
		No stroke treatment	3.05	1.96	4.75	<.0001	.33	.14	.78	.0116

†*P*-value from the Fine and Gray proportional hazard distribution regression model.

^a^Univariate model hazard ratios (HR) and 95% confidence intervals (CI) for each competing risk.

### Multivariate modelling

The covariates considered for the multivariate model in this study include age, gender, admission source, stroke diagnosis at admission, stroke type, stroke history, cancer and dementia comorbidities, code status at admission, and GCS at admission. However, other covariates such as, NIHSS, ASPECTS, severe stroke, and treatment classification were not included in the multivariate model for various reasons such as too many missing values, was confounded with other covariates or lack of significance in the univariate model.

The multivariate model with the selected covariates (n = 231, Table 3) revealed that only the GCS coma-related category was significantly associated with a higher incidence of death (*P* = .04). There were no significant covariates associated with the competing risk of PMC. Although the direction of hazard ratios (HRs) for both competing risks remained consistent with the univariate results, the statistical power was reduced due to missing covariate values. Additionally, some demographic and clinical characteristics showed additional confounding effects in relation to the competing risk outcomes (ie, reducing the strength of the HR). In summary, the multivariate model analysis suggests that the GCS coma-related category is a significant predictor of death, even after adjusting for various covariates. Further research with larger sample sizes and complete data sets may provide a more robust understanding of the relationships between these covariates and both competing risk outcomes.

**Table 3.** Multivariate Fine and Gray Model – Competing Risk Analysis.

	N = 231	Value	Death in Hospital				Palliative Care Consult			
			HR^a^	95%CI		*P* ^†^	HR^a^	95%CI		*P* ^†^
Age (per year)		Older age	.99	.98	1.00	.1713	1.03	1.00	1.06	.0850
Gender (ref male)		Female	.86	.62	1.20	.3850	1.66	.93	2.96	.0878
Admission source (ref home in the community)		Acute care hospital	1.38	.99	1.92	.0614	.54	.27	1.10	.0913
		Other	.91	.41	2.05	.8244	1.27	.49	3.27	.6278
Stroke at admission (ref yes)		No	.67	.40	1.10	.1104	1.56	.80	3.05	.1906
Stroke type (ref hemorrhagic stroke)		Ischemic stroke	.78	.56	1.08	.1362	1.35	.77	2.39	.2985
Stroke history (ref first stroke)		Recurrent stroke	.95	.64	1.42	.7981	1.13	.58	2.19	.7268
Cancer comorbidity (ref no)		Yes	.92	.60	1.40	.6900	1.39	.78	2.50	.2637
Dementia comorbidity (ref no)		Yes	.66	.38	1.16	.1500	1.12	.55	2.26	.7615
Code status at admission (ref full code)		DNR	1.28	.93	1.76	.1303	1.12	.64	1.95	.6944
GCS at admission (ref 9-15)		3-8 - coma	1.40	1.02	1.92	.0394	.61	.34	1.08	.0893

†*P*-value from the Fine and Gray proportional hazard distribution regression model.

^a^Univariate model hazard ratios (HR) and 95% confidence intervals (CI) for each competing risk.

## Discussion

This study underscores a critical gap in the integration of palliative care in stroke management with potential relevance for regional stroke centers. Palliative care, traditionally aligned more with chronic illnesses, must adapt to the acute and unpredictable trajectory of stroke to effectively meet patient needs from the onset of care. Despite recognized benefits such as enhanced quality of life and better symptom management, our findings highlight a significant delay in the initiation of palliative care consultations, often reserved for end-of-life stages rather than being integrated as a part of initial stroke care. This delay may reflect a persistent underutilization of palliative resources or a lack of protocols that integrate palliative approaches early in stroke management pathways. This study identified several clinical characteristics to better understand and integrate palliative care services into stroke treatment among a cohort of severe stroke patients admitted to a regional stroke centre that subsequently died. This information may help inform stroke care teams of those patients who are at risk for a higher incidence of death and a lower incidence of a PMC - to offer families palliative care consults early in care given the time to death identified in our study was a median of 6 days. Our study adds to the literature by identifying clinical characteristics among cohorts of stroke patients at risk for a low incidence of PMC. Our findings may prompt further review of local practice consideration and advocacy for change that supports early inclusion of palliative care in stroke treatment protocols, potentially mirroring the more proactive approaches seen in oncology settings.

The results highlight that only 28.8% of these patients received a PMC, and the median time to consultation was 6 days. Univariate analyses revealed that older age, female gender, absence of stroke diagnosis at admission, ischemic stroke, and comorbidities of cancer or dementia were associated with a higher incidence of PMC. Conversely, admission from an acute care hospital and lower Glasgow Coma Scale (GCS) scores were associated with a lower incidence of PMC. In the multivariate model, only lower GCS scores remained significantly associated with a higher incidence of death, while no factors were significantly associated with PMC. In these cases, patients with a GCS coma classification are unable to partake in GOC conversations. Turning to their Substitute Decision Makers (SDMs) to engage with PMC can require time which can be a competing factor for patients getting a PMC given that the time to death identified in our study was a median of 6 days. Factors can include developing a therapeutic relationship with the stroke team, the time families need to process the severity of the stroke, and the time families need to make care decisions. The fact that our study highlights that some patients with GCS coma classification did not receive PMC may relate to palliative care practices in acute stroke being a more complex, nuanced process - not a simple or linear one.

Thus, this combination of results may be explained by the fact that end-of-life measures tend to be offered to these patients early in admission. These findings underscore the need for improved integration of palliative care in the management of acute stroke patients, particularly those with severe neurological deficits, to ensure timely access to comprehensive, patient-centered care throughout the illness trajectory.

The results observed in this study may have multiple underlying causes. One potential explanation for the higher incidence of PMC in females is their tendency to use healthcare in a fundamentally different manner than males, which has been well-documented in the literature. Societal and cultural factors, such as gender-specific healthcare beliefs, and different communication styles may influence healthcare-seeking behavior. Our findings are in keeping with other studies that demonstrate women often take on help-seeking roles in end-of-life discussions and actively engage in doctor-patient communication to develop a more wholesome understanding of their illness state.^17,18^ Similarly, a multicenter study by Busquet-Duran et al examined gender differences in palliative care patients and found that females often relied on spirituality, a fundamental pillar of palliative care, to cope with life-threatening illnesses.^19^ Therefore, it is plausible that the female stroke patients in our study may have both requested and relied on the central tenets of the palliative care philosophy and as such, were more likely to receive a PMC.

Stroke patients transferred from a peripheral acute care hospital were associated with a lower incidence of PMC compared to patients directly admitted to patients admitted to SHSC directly from the community. The delivery of effective palliative care requires a therapeutic relationship between the patient and the health care team, which is widely supported in literature .^20-22^ However, the transfer of patient care among healthcare practitioners may hinder or delay the development of a therapeutic relationship, reducing the likelihood of a palliative care consultation before the patient’s death. It is also important to note that patients admitted from external acute hospitals may differ fundamentally from those admitted from the community, in demographics not captured by this study. For instance, if more patients were mechanically ventilated when transferred, they would be less likely to participate in discussions regarding palliative care. This explanation is supported by our own findings where those stroke patients with GCS coma classification and admission from acute care hospitals were less likely to receive a PMC. A retrospective multi-center study by Comer et al found that although PMCs were significantly associated with greater stroke severity, those stroke patients severely disabled enough to require percutaneous feeding or tracheostomy were no more likely to receive a PMC.^23^ Thus, these findings may also demonstrate missed opportunities for PMC for those acute stroke care patients with such severe disabilities whose families will likely benefit from goals-of-care discussion and support from the palliative care team.

Our study also found that older patients and those with pre-existing comorbidities such as cancer and dementia were more likely to receive a PMC. The results showed that older age was associated with a higher incidence of PMC in univariate analyses. This finding does not necessarily reflect a negative attitude towards older people, but rather may be explained by several factors. Older patients carry a higher burden of medical comorbidities than younger patients and increasing frailty scores are known to correlate with advanced age. Both burden of comorbidities and frailty have been associated with increased palliative care utilization.^24-26^ It is also often seen as more appropriate from a societal view to pursue a holistic care approach such as palliative care in the elderly where advanced care planning and discussion of goals of care, substitute decision makers, power of attorney, and code status naturally become more relevant.^27,28^ A combination of these factors may explain the increased incidence of palliative care consultation in the stroke patients of this study. Additionally, many of these potential comorbidities that the older patients may have had in this study include cancer and dementia which are both diagnoses that are well-established and integrated in palliative care. As a result, patients and their families who have been coping with these diagnoses may have a greater understanding of end-of-life philosophies related to palliative care and may have already experienced a palliative care approach previously. They may have been able to utilize this knowledge and experience with palliative care during these admissions and expressed a desire to integrate palliative care in their stroke treatment approach or in combination with palliative care they already have in place for their cancer or dementia diagnosis. Indeed, our recent survey of stroke physicians found that many noted the palliative care team is more likely to be consulted in acute severe stroke patients when the patient’s family has adequate education regarding palliative care.^29^

No stroke diagnosis at admission to hospital and ischemic stroke were noted to be associated with an increased incidence of PMC. Patients who were noted to have a stroke after admission to hospital may have had a higher burden of comorbidities due to already being admitted to hospital for another active issue. This higher burden of medical care may have played an influence on the desire to pursue palliative care. Patients with ischemic stroke were also noted to be more likely to obtain a PMC compared to those with hemorrhagic stroke. Ischemic strokes are known to occur more frequently in older populations than its hemorrhagic counterpart.^30^ As previously discussed, older age may increase the incidence of palliative care through a variety of mechanisms.

Furthermore, in multivariate modelling, GCS 3-8, designated as coma GCS, was noted to non-significantly decrease the incidence of palliative care consultation. Typically, stroke patients presenting with a very depressed GCS herald an unfavorable outcome with a high initial mortality. Patients with a DNR status were less likely to get a PMC. Patients who have an established DNR order on admission may have already had a goals of care or palliative care discussion prior to their acute stroke admission. Therefore, another PMC may not have been thought to be indicated by the clinical team. This raises the possibility of a barrier to understanding the larger scope of support palliative care can offer to patients and their families facing serious illness such as acute stroke. Also, results may be explained by the fact that end of life measures tend to be offered to these patients early in admission, and tend to occur ahead of palliative care consultation.^23,30^

Our univariate modelling also found that stroke patients who received a therapeutic intervention were less likely to get a PMC. This finding may illustrate the unfortunate misconception of palliative care in acute stroke care and consequent missed opportunities for care whereby those patients who underwent an intervention may seem to have a better prognosis and thus, did not seem to need a palliative care consultation. Yet, the core of palliative care is disease and prognosis agnostic and is based on illness severity. Thus, there is always an opportunity for a palliative approach to care in stroke care where the risk of morbidity and mortality is incredibly high and thus, palliative care discussions are warranted.

## Limitations

The purpose of the study was to investigate clinical characteristics associated with the integration palliative care in this stroke population through a retrospective chart review and did not investigate family and physician related factors to facilitating a PCC. While this work contributes to the discussion and understanding of the clinical characteristics identified with the incidence of a PCC, future research could investigate family and physician related factors among this patient cohort. In a multicenter cohort that included patients hospitalized with severe ischemic stroke, the study found that most patients with severe stroke did not receive a PMC, even among those who died in hospital, which is consistent with our findings.^23^

There are some limitations to this study. Firstly, our study is largely exploratory in nature and is based in an academic, acute stroke centre in which palliative care services are well-established. As such, there is limited generalization to other stroke settings including community sites without healthcare trainees and established palliative care services. For instance, palliative care services can be streamlined into both generalist palliative and specialist palliative care services where the latter may be initiated in more nuanced situations such as patient disposition complexity and complex pain management. Moreover, in-hospital stroke teams are often interdisciplinary in nature with social work available to provide various supports including bereavement services.^31^It is plausible then, that the stroke care physicians in this study may have been comfortable conducting generalist palliative care and thus, did not formally initiate a PMC.

In addition, due to the retrospective design of this study, the specific causal relationship between PMC and important outcomes cannot be delineated. Future studies should explore the causal nature between the PMC and these outcomes through prospective study design. This would also allow for better capture of important covariates and would improve statistical power of multivariate models. Also, future studies could consider including patients who survived their stroke. Moreover, future studies should also be conducted in various stroke care settings potentially through the use of mixed methods whereby qualitative data, through interviewing healthcare practitioners and patient family members, can provide further rich insight. It may also be of interest for future studies to consider how clinical characteristics may differ in early vs late PMCs in stroke care. Additionally, given that those patients who received an intervention were less likely to receive a PMC in univariate analysis, it may also be of interest to have garnered other significant clinical characteristics in our data collection including prognosis. Finally, given recent times, it may also be of interest to further explore the impacts of the global COVID-19 pandemic on PMC utilization in acute stroke care. The study period (January 2nd, 2016, to February 28th, 2020) overlaps with the early stages of the pandemic, which may have influenced healthcare practices and resource allocation. Exploring how the pandemic affected PMC utilization and patient outcomes could provide valuable insights for future research and policy development.

## Conclusion

Given the major impact of stroke worldwide, there remains a significant need to better understand and integrate palliative care services into stroke treatment. Our study identifies several clinical characteristics associated with palliative medicine consultations (PMC) in stroke care and specifically demonstrates that older age, being female, no stroke diagnosis at admission, ischemic stroke and comorbidities of cancer or dementia were associated with higher incidence of PMC, while admission from an acute care hospital and a GCS coma classification were associated with a lower incidence of PMC. When adjusting for demographic and clinical characteristics, our study also identifies the GCS coma-related category as the only significant factor associated with a higher incidence of death, but it is also non-significantly associated with a low incidence of PMC.

Ultimately, these results highlight a missed opportunity for palliative care in stroke patients where palliative care services are readily accessible for other conditions such as cancer and dementia which act as immediate triggers for a PMC. Our results show that there is a grave disparity in stroke patients who do not present with such comorbidities and thus, unfortunately are unlikely to get access to palliative care services. As such, more prospective studies conducted in various stroke care settings are needed to better elucidate and optimize the use of PMC in stroke care.
